# High-Level Expression, Purification and Large-Scale Production of l-Methionine γ-Lyase from *Idiomarina* as a Novel Anti-Leukemic Drug

**DOI:** 10.3390/md13085492

**Published:** 2015-08-21

**Authors:** Kui-Ying Huang, Hai-Yan Hu, Yan-Lai Tang, Feng-Geng Xia, Xue-Qun Luo, Jian-Zhong Liu

**Affiliations:** 1 South China Sea Bio-Resource Exploitation and Utilization Collaborative Innovation Center, School of Life Sciences, Sun Yat-sen University, Guangzhou 510275, China; E-Mail: qiushuixian2004@163.com; 2 Guangzhou Institute of Microbiology, Guangzhou 510663, China; E-Mails: huhaiyansysu@163.com (H.-Y.H.); xiafenggeng@163.com (F.-G.X.); 3 Department of Pediatrics, The First Affiliated Hospital, Sun Yat-sen University, 58, Zhong Shan Second Road, Guangzhou 510080, China; E-Mail: tangyanlai@126.com

**Keywords:** l-Methionine γ-lyase, purification, production, enzyme, leukemia, anti-leukemic drug, cell proliferation, apoptosis

## Abstract

l-Methionine γ-lyase (MGL), a pyridoxal 5′-phosphate-dependent enzyme, possesses anti-tumor activity. However, the low activity of MGL blocks the anti-tumor effect. This study describes an efficient production process for the recombinant MGL (rMGL) from *Idiomarina* constructed using the overexpression plasmid in *Escherichia coli* BL21 (DE3), purification, and large-scale production. The enzyme produced by the transformants accounted for 53% of the total proteins and accumulated at 1.95 mg/mL using a 500 L fermentor. The enzyme was purified to approximately 99% purity using a high-pressure mechanical homogenizer and nickel (Ni) Sepharose 6 Fast Flow (FF) chromatography. Then, the enzyme was polished by gel filtration, the endotoxins were removed using diethyl-aminoethanol (DEAE) Sepharose FF, and the final product was lyophilized with a vacuum freeze dryer at −35 °C. The specific activity of rMGL in the lyophilized powder was up to 108 U/mg. Compared to the control, the enzyme significantly inhibited cellular proliferation in a concentration-dependent manner as tested using the MTS (3-(4,5-dimethylthiazol-2-yl)-5-(3-carboxymethoxyphenyl)-2-(4-sulfophenyl)-2*H*-tetrazolium) assay and induced cellular apoptosis as analyzed by Annexin V-fluorescein isothiocyanate (FITC) with fluorescence-activated cell sorting (FACS) in leukemia cells. This paper demonstrated the cloning, overexpression, and large-scale production protocols for rMGL, which enabled rMGL to be used as a novel anti-leukemic drug.

## 1. Introduction

l-Methionine γ-lyase (MGL), a pyridoxal 5′-phosphate-dependent enzyme, catalyzes the γ-elimination of l-Methionine to generate α-ketobutyrate, methanethiol, and ammonia as well as the α,β-replacement and β-elimination of *S*-substituted l-cysteines [1]. MGLs have been discovered, isolated, and purified from bacteria, especially from *Pseudomonas ovalis*. The MGL gene from *Pseudomonas putida* was cloned and expressed at low levels in *Escherichia coli* [2,3,4]. 

To produce the enzyme on a large scale, a scale-up production process for the recombinant MGL (rMGL) was established, which contained a heat step, two diethyl-aminoethanol (DEAE) Sepharose Fast Flow (FF) ion-exchanges, and one ActiClen Exox endotoxin-affinity chromatography column [5]. Additionally, another efficient production process was built-in by plasmid overexpression and cultivation and crystallization on an industrial scale [6]. Although their processes could be used to produce rMGL on a large scale, the activity of the enzyme was still low. 

Our research team has identified a novel MGL gene from *Idiomarina* [7]. The MGL gene has been cloned and overexpressed in *E. coli* using the PET-28a (+) vector. The efficiency of the rMGL was verified to catalyze the lysis of l-Methionine and dl-homocysteine. We not only need to produce an active and useful rMGL, but we also need to build a production process that will enable large-scale production of endotoxin-free rMGL for therapeutic use. 

Many human tumor cells require l-Methionine as an essential amino acid for growth [8]. l-Methionine-dependent tumor cells cannot divide and proliferate when there is a lack of l-Methionine [9,10]. However, normal cells are relatively resistant to l-Methionine depletion [11]. Therefore, depleting l-Methionine is a safe and effective cancer treatment. This cancer treatment strategy has previously been reported [12,13,14]. *In vitro* and *in vivo* studies of the anti-tumor activity of rMGL have indicated that many types of human tumors, such as lung, brain, colon, prostate, kidney, and melanoma, were sensitive to rMGL. Meanwhile, no toxicity has been detected in animal experiments [6,15]. Recently, acute leukemia failed to achieve long-term disease-free survival [16]. Thus, we need to find novel and less toxic drugs for the treatment of patients with acute leukemia. We found that flavokawain B might be a possible therapeutic drug for patients with acute lymphoblastic leukemia (ALL). The potential role of rMGL as an anti-tumor drug in leukemia needs to be tested. 

In this research, we showed an effective large-scale production process for rMGL constructed using an overexpression plasmid in *E. coli* BL21 (DE3), followed by purification and large-scale production. We demonstrated the anti-leukemic effect of rMGL in ALL and acute myeloid leukemia (AML) cell lines. We showed that rMGL was effective in inhibiting leukemic cell proliferation and promoting cellular apoptosis. Altogether, our results indicated that rMGL might be a possible therapeutic drug for patients with acute leukemia.

## 2. Results 

### 2.1. Large-Scale Fermentation for rMGL Production

**Figure 1:**
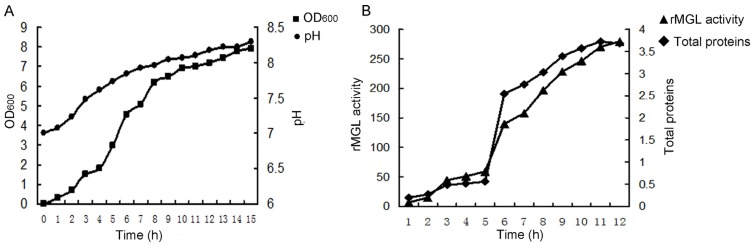
The time course of *E. coli* BL21 (DE3) fermentation in a 300 L production culture using a 500 L fermentor. (**A**) The cell growth (OD_600_) and the pH values during the fermentation process; (**B**) The rMGL activity (U/mL) and total proteins (mg/mL) at different times.

Fermentation was conducted in a 500 L fermentor using 300 L of production medium. Cell growth increased linearly during 4–10 h of cultivation with the specific growth rate during 0.85–1 h at logarithmic growth phase. The pH of the medium increased from 7.0 to 8.3 during the fermentation phase (Figure 1A). As shown in Figure 1B, the rMGL activity increased up to 279.5 U/mL at 12 h. The total protein weight reached 3.68 mg/mL at the end of the fermentation. Cell growth increased linearly during 4–10 h of cultivation with the specific growth rate during 0.85–1 h at the logarithmic growth phase.

### 2.2. SDS-PAGE Analysis of Purified rMGL

**Figure 2:**
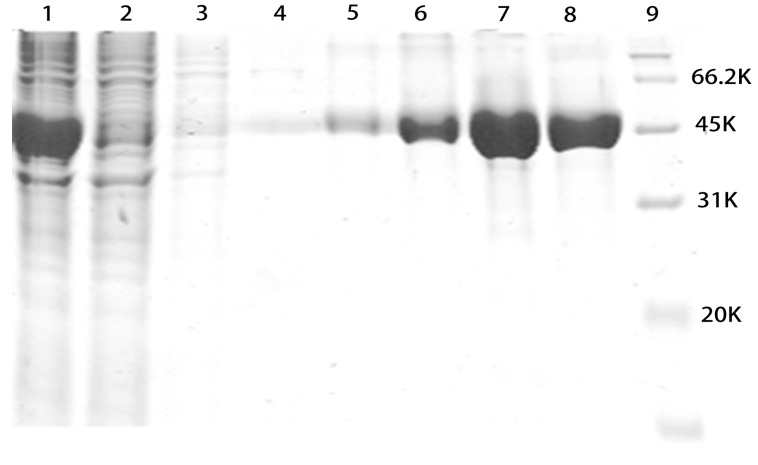
SDS-PAGE analysis of the samples. Lane 1, crude enzyme; Lane 2, flow through; Lane 3, 40 mM imidazole elution; Lane 4, 80 mM imidazole elution; Lane 5, 160 mM imidazole elution; Lane 6, 200 mM imidazole elution; Lane 7, 300 mM imidazole elution; Lane 8, 400 mM imidazole elution; Lane 9, marker.

After fermentation in the production culture, the cells were harvested by centrifugation and disrupted using a homogenizer. The cellular debris was removed by centrifugation. Crude enzyme was collected and applied to a Ni Sepharose 6 FF column. Unwanted proteins were washed away with 80 mM imidazole buffer. rMGL was eluted stepwise with buffers containing 160, 200, 300, and 400 mM imidazole. The elution fractions were collected and tested by sodium dodecyl sulfate polyacrylamide gel electrophoresis (SDS-PAGE). As shown in Figure 2, all of the elution fractions corresponded to 46 kDa on the SDS-PAGE. 

### 2.3. HPLC Analysis of Purified rMGL

**Figure 3:**
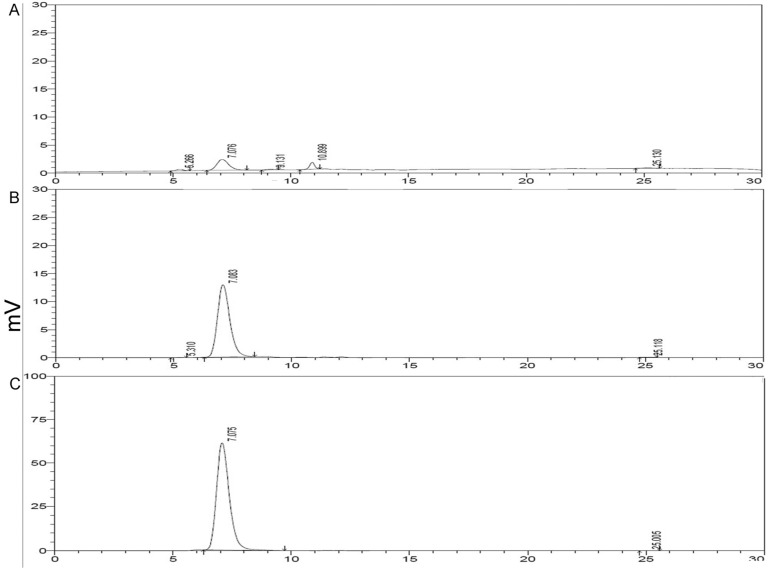

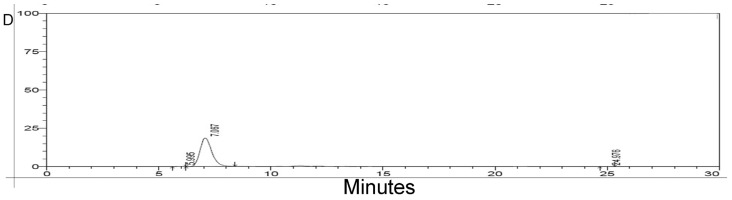
HPLC analysis of purified rMGL. (**A**) rMGL eluted with 160 mM imidazole buffer; (**B**) rMGL eluted with 200 mM imidazole buffer; (**C**) rMGL eluted with 300 mM imidazole buffer; (**D**) rMGL eluted with 400 mM imidazole buffer.

As shown in Figure 3, the HPLC analysis demonstrated a major peak with a retention time of 7.08 min. The purity calculation indicated that the purity of rMGL eluted with 160 mM imidazole was 69.07%, while the purities of rMGL eluted with 200, 300, and 400 mM imidazole buffer were 99.34%, 99.76%, and 99.92%, respectively.

### 2.4. Lyophilization of rMGL

**Figure A1:**
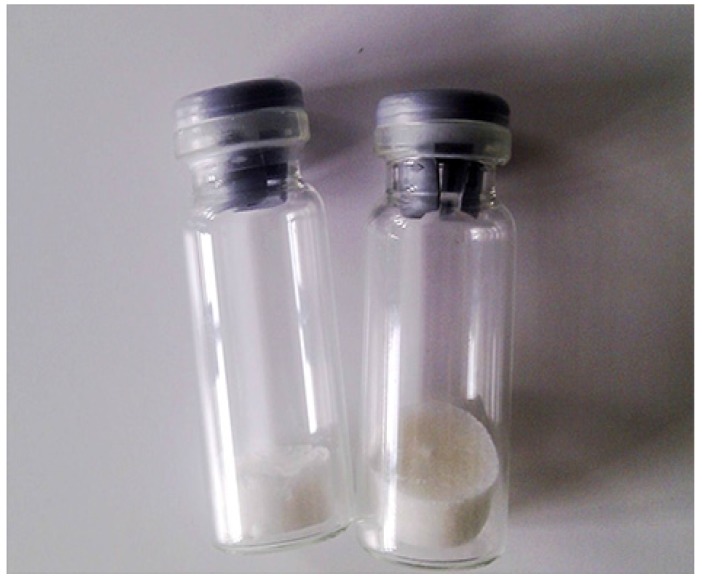
rMGL powder after vacuum freeze drying.

As shown in Figure A1, purified rMGL was lyophilized after desalinization and after the endotoxin was eliminated. The enzyme products are white powder. As shown in Table 1, rMGL purification is summarized. The specific activity of rMGL in lyophilized powder was 107.95 U/mg. 

**Table 1:** rMGL purification.

Step	Volume (L)	Activity (U)	Protein (g)	SA * (U/mg)	Yield (%)
HG *	30	75,600,000	1104	68.47	90
Ni-FF *	10	71,820,000	567.57	126.54	85.50
DS * and CO *	2	66,792,600	544.86	122.59	79.52
EN *	2	60,113,340	506.72	118.63	71.56
Drying *	--	54,703,139	506.72	107.95	57.25

* SA indicates specific activity; HG indicates homogenization; Ni-FF represents Ni Sepharose 6 Fast Flow; DS indicates desalination; CO indicates concentration; EN represents endotoxin removal; Drying represents vacuum freeze drying.

### 2.5. Comparison of rMGL Production from Various Strains 

As shown in Table 2, rMGL production from various strains is summarized. 

**Table 2:** Summary of rMGL production from various strains.

*E. coli* Strain	Plasmid	F * (L)	rMGL (mg/mL)	Content	Activity (U/mg)
MV1184 [4]	pYH103	--	--	3.7	3.7
BL21(DE3) [5]	pAC-1	0.8	1	14	3
JM109 [6]	pMGLTrc03	300	2.1	43	57
BL21(DE3) *	pET-mgl	500	1.95	53	107.95

* F indicates fermentor; BL21(DE3) was used in this study.

### 2.6. rMGL Inhibits the Proliferation of Leukemia Cell Lines

**Figure 4:**
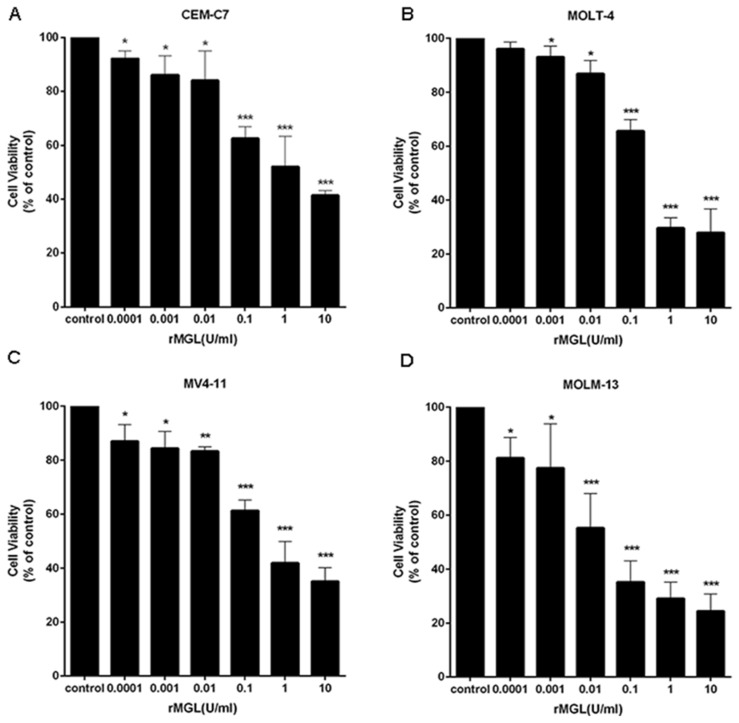
rMGL suppresses the viability of leukemia cells CEM-C7 (**A**), MOLT-4 (**B**), MV4-11 (**C**) and MOLM-13 (**D**). The cell lines were exposed to different concentrations of rMGL for 48 h, and cell viability was evaluated using the MTS assay. The data are presented as the means ± s.d. of three independent experiments.

To determine the effects of rMGL on the proliferation of human leukemia cells, we tested the viability of leukemia cell lines exposed to various concentrations of rMGL for 48 h using the MTS assay. As shown in Figure 4, rMGL significantly suppressed the proliferation of leukemia cell lines in a dose-dependent manner.

### 2.7. rMGL Induces Apoptosis of Leukemia Cell Lines

**Figure 5:**
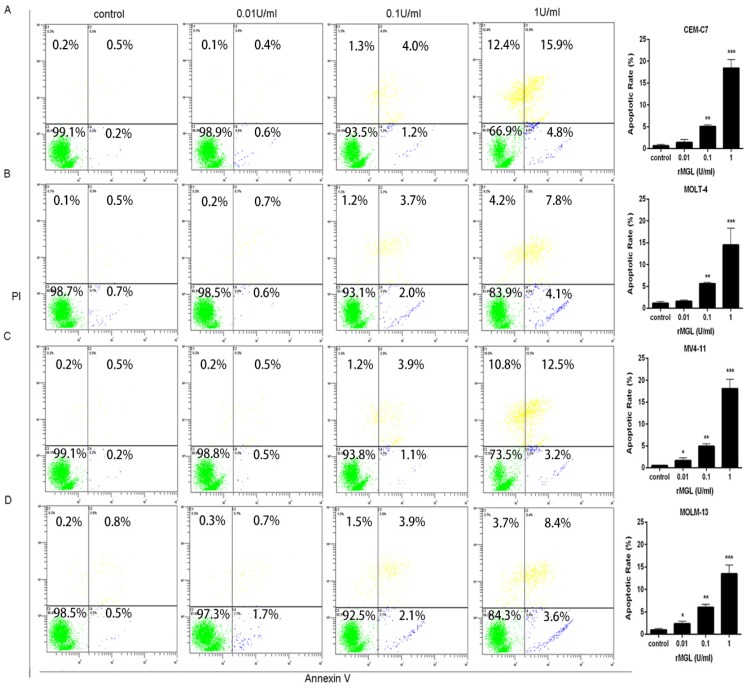
rMGL promotes cell apoptosis. The apoptotic rate was tested using Annexin V-FITC/PI staining and flow cytometry in the leukemia cell lines CEM-C7 (**A**), MOLT-4 (**B**), MV4-11 (**C**) and MOLM-13 (**D**) exposed to 0.01, 0.1, and 1 U/mL rMGL for 48 h. The results are presented as the means ± s.d. of three independent experiments.

To examine the effects of rMGL on the apoptosis of leukemia cells, we tested the apoptotic rates of leukemia cells exposed to 0.01, 0.1, and 1 U/mL rMGL for 48 h by flow cytometry. rMGL treatment resulted in obvious increases in both early and late apoptotic populations in leukemia cells compared with the control group (Figure 5).

## 3. Discussion 

**Figure A2:**
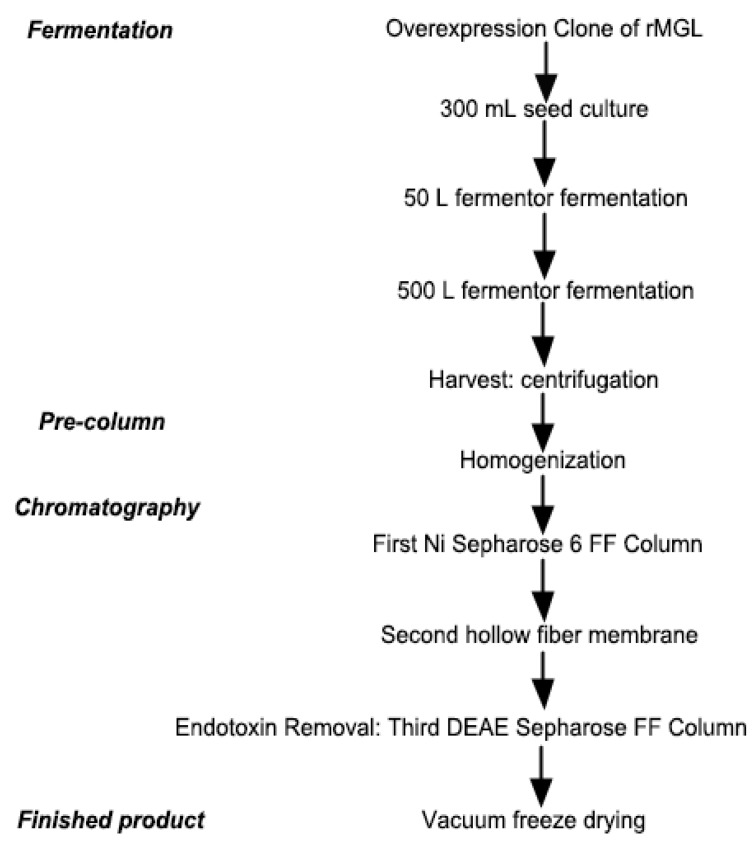
The flowchart for large-scale production of rMGL.

Methionine is necessary for cancer cells compared to normal cells both *in vitro* and *in vivo* [17,18,19,20,21,22]. Based on previous reports suggesting that methioninase has anti-carcinogenic properties on some types of cancers, methioninase may be used to target some malignancies with minimal toxicity [23,24,25]. Therefore, the large-scale production of recombinant methioninase was necessary. In this study, we showed an industrial manufacturing process of rMGL by high-level gene expression, large-scale fermentation, purification, and lyophilization (Figure A2). The results revealed that cell growth markedly increased during large-scale fermentation. Additionall, rMGL activity reached a maximum at 279.5 U/mL (Figure 1). After fermentation in the production culture, the analysis of purified rMGL tested by SDS-PAGE and HPLC showed that the purities of rMGL eluted with 200, 300, and 400 mM imidazole buffer were all more than 99% (Figure 3). After the purified rMGL was desalinated and the endotoxin eliminated, the final product was lyophilized as a white powder (Figure A1). High-level expression and the purification process of the enzyme provided a cost-effective manufacturing process. Table 2 summarizes the comparison of rMGL expressed in various transformants. We were able to obtain 1.95 g/L of rMGL as a soluble form using a 500 L fermentor. The content reached 53% of the total proteins in the soluble fraction in this research. In this study, the specific activity of rMGL was 107.95 U/mg, which was much greater than values reported by Tan *et al*. [5] and Takakura *et al*. [6]. 

The majority of AML patients who received intensive chemotherapy did not achieve complete remission. The relapse rate was high, and the overall five-year survival rate was almost 25%. Patients who were older than 60 years had a poorer prognosis [26,27]. Therefore, it was very important to discover and identify new therapeutic methods for the majority of AML patients. ALL is the most common childhood malignancy and comprises nearly one-third of all pediatric malignancies [28]. Although modern treatment strategies achieved improvement, nearly 20% of the children with ALL relapsed or died [29,30,31,32]. The capacity of leukemic cells to proliferate quickly and survive has been associated with disease progression [33]. Therefore, acting on anti-proliferative signals or pro-apoptotic signals may constitute novel therapies for acute leukemia [34,35,36]. Evidence suggests that methioninase has anti-tumor properties in some types of cancers [37,38]; we hypothesized that rMGL might have an anti-leukemic effect on leukemia cells. 

In this study, we investigated the anti-leukemic activities of rMGL in ALL and AML cell lines. The proliferation test was conducted, and we observed that rMGL significantly inhibited leukemia cell growth (Figure 4). The results showed that rMGL significantly promoted leukemia cell apoptosis in a dose-dependent manner (Figure 5). Several reports have described the association between cellular apoptosis and cancer [39,40], and apoptosis induced by rMGL could be developed for the management of acute leukemia. 

## 4. Materials and Methods

### 4.1. Materials

Nickel (Ni) Sepharose 6 FF and DEAE Sepharose FF were obtained from Pharmacia Biotech Inc. (Piscataway, NJ, USA). Phenylmethanesulfonyl fluoride (PMSF) and imidazole were purchased from Sigma (St. Louis, MO, USA). Peptone was purchased from OXOID (Basingstoke, Hampshire, UK), and yeast extract was purchased from Angel Yeast (Angel Yeast Co., Ltd, Yichang, China). Other reagents used in this research were analytical reagent grade or the highest grade commercially available. 

### 4.2. Bacteria Strains and Media

The MGL gene from the deep-sea sediment metagenomic library was cloned and expressed in *E. coli* with vector PET-28a (+) containing the T7 promoter (Merck Millipore Corporation, Darmstadt, Germany). The expression conditions of the rMGL protein have been optimized. The seed medium included peptone (10 g/L), yeast extract (5 g/L), and NaCl (5 g/L). The production medium contained glycerol (4 g/L), peptone (8 g/L), yeast extract (8 g/L), and NaCl (6 g/L).

### 4.3. Large-Scale Production of rMGL

A flowchart for large-scale production of rMGL is shown in Figure A2.

#### 4.3.1. Fermentation

(1) First preculture: The rMGL strain was maintained as a frozen vegetative stock at −80 °C in 20% glycerol. A total of 30 μL of bacteria from a vial was inoculated into 300 mL of fresh seed medium containing kanamycin and was shaken at 37 °C overnight. 

(2) Second preculture: The first preculture broth was transferred to a 30 L seed medium containing 25 μg/mL kanamycin in a 50 L fermentor and was cultivated at 37 °C under agitation at 200 rpm with aeration at a rate of 25 L/min for 4 h.

(3) Production culture: The second preculture broth was transferred to 300 L of medium containing 25 μg/mL kanamycin in a 500 L fermentor under agitation at 250 rpm with aeration at a rate of 25 L/min for 3 h. Then, the isopropyl-β-d-thiogalactopyranoside (IPTG) was added at a final concentration of 0.1 mM and incubated for another 3 h at 25 °C before harvesting.

#### 4.3.2. Preparation of the Crude Enzyme 

After fermentation for 6 h in the production culture, the bacterial cells were harvested by centrifugation at 8000 rpm at 4 °C for 15 min and concentrated as a cell suspension. The cell suspensions were diluted with ice-cold extraction solution (100 mM potassium phosphate (pH 7.4) containing 40 mM imidazole, 0.5 M NaCl, 0.01% dithiothreitol, 0.1 mM PMSF, and 10 μM pyridoxal phosphate (PLP)). Then, the cell suspensions were disrupted twice with passages through a high-pressure mechanical homogenizer (Invensys Systems APS, Albertslund, Denmark) at 10,000 psi. The cellular debris was removed by centrifugation at 18,000 rpm at 4 °C for 15 min. The recombinant enzyme that was produced intracellularly in a soluble form was accumulated in the clarified supernatant. The pH of the concentrated crude enzyme was adjusted to 7.4 with 10 M NaOH. 

#### 4.3.3. Chromatographic Conditions 

(1) First column: Ni Sepharose 6 FF (Pharmacia Biotech Inc., Piscataway, NJ, USA). The first column was 100 mm in diameter and 30 cm in height with a volume of 2000 mL of Ni Sepharose 6 FF. The column was washed with equilibration buffer (100 mM sodium phosphate, 80 mM imidazole, 0.5 M NaCl, pH 7.4). A total of 30 L of crude enzyme supernatant was applied to the column. After loading, the column was prewashed with equilibration buffer for approximately five volumes until the OD_280_ dropped below 0.01. The protein was then eluted stepwise with equilibration buffer containing 80, 160, 200, 300, and 400 mM imidazole. The elution fractions containing the enzyme were collected in 10-L volumes.

(2) Desalination and concentration: A hollow fiber membrane (molecular weight cut-off of 20 kDa, Xubang Membrane Equipment Co. Ltd, Beijing, China) was used for desalination. After 10 L of the solution containing enzyme was applied to the hollow fiber column, which had been equilibrated with 5 mM sodium phosphate buffer (pH 7.4), the enzyme was eluted with the same buffer at a flow rate of 30 L/h. The enzyme fractions were desalted and concentrated to 2 L. Then, 10 mM PLP was added to the solution at a final concentration of 10 μm. 

(3) Endotoxin removal: To eliminate endotoxin, 2 L of the concentrated enzyme solution was applied on a 1000 mL DEAE Sepharose FF column (Pharmacia Biotech Inc., Piscataway, NJ, USA). The enzyme was eluted with 5 mM sodium phosphate buffer (pH 7.4) at a flow rate of 10 mL/min. The enzyme fractions, identified by their yellow color, were collected until the OD_280_ dropped below 0.1. 

#### 4.3.4. Finished Product

Following the above step, the enzyme solution was sub-packaged into a 10 mL Cillin glass vial (Jiangsu Huayue Pharmaceutical Glass Co., Ltd. Danyang, China) at 2 mL per vial. All of the samples were then put into a lyophilizer to be frozen at a speed of 1 °C/min until reaching −35 °C. After they were fully frozen, the samples were dried at −28 °C under vacuum conditions, followed by desorption drying at 25 °C.

### 4.4. Analysis of rMGL

#### 4.4.1. Biomass

Cell growth during fermentation was monitored by measuring the OD_600_ with a spectrophotometer (UV-1600; Shimadzu, Kyoto, Japan). The biomass was determined with dry cell weight according to the method of Takakura *et al*. [6]. A total of 5 mL of the culture broth was centrifuged (8000× *g*, 10 min) in a pre-weighted glass test tube. Then, the cell pellet was dried at 90 °C until a constant weight was achieved. 

#### 4.4.2. Protein Concentration

The protein concentration was determined using a Bradford Assay Method with a Bradford Protein Assay Kit (Sangon, Shanghai, China) with bovine serum albumin as the standard protein.

#### 4.4.3. Activity Assay

The rMGL activity was determined by the method of Tanaka *et al*. [3]. One unit of rMGL was defined as the amount of enzyme that produced 1 μmol of α-ketobutyrate per minute at an infinite concentration of l-Methionine. The specific activity was represented as the enzyme activity in units per milligram of protein. 

#### 4.4.4. HPLC

An Agilent high-performance liquid chromatography (HPLC) 1200 system (Agilent Technologies, Palo Alto, CA, USA) with a Supelco Progel TSK G3000SW column (30 cm × 7.5 mm) (Supelco, Bellefonte, PA, USA) was used for this HPLC experiment. A total of 20 μL of sample was loaded and eluted with elution solution (0.1 M sodium phosphate buffer, 0.1 M sodium chloride, and pH 7.0) at a flow rate of 1.0 mL/min.

### 4.5. Cell Lines and Cell Culture

CEM-C7, MOLT-4, MV4-11, and MOLM-13 cell lines were purchased from the American Type Culture Collection (ATCC, Manassas, VA, USA). All of the cells were routinely grown in a humidified atmosphere with 5% CO_2_ at 37 °C and were maintained in Roswell Park Memorial Institute (RPMI) 1640 medium supplemented with 10% fetal bovine serum (HyClone, Logan, UT, USA). The characteristics of the cell lines are detailed in Table 3.

**Table 3:** Characteristics of the cell lines used in this study.

		Cell Line		
Characteristics	CEM-C7	MOLT-4	MV4-11	MOLM-13
Cell type	T-ALL	T-ALL	AML	AML
Primary site	PB *	PB	PB	PB
IC_50_ * (U/mL)	1.70	0.30	0.64	0.03

* PB indicates peripheral blood; IC_50_ represents half-inhibitory concentration and was calculated using GraphPad Prism 6 software with triplicate values from MTS (3-(4,5-dimethylthiazol-2-yl)-5-(3-carboxymethoxyphenyl)-2-(4-sulfophenyl)-2H-tetrazolium) assays.

### 4.6. Cell Proliferation Assay

The anti-leukemic activities were tested using an MTS assay (Promega, Madison, WI, USA). A total of 1 × 10^4^ leukemia cells per well were treated with different concentrations of rMGL. After 48 h of incubation, the cell viability was determined using an MTS assay according to the manufacturer’s instructions. 

### 4.7. Apoptosis Assay

The apoptotic rate was tested using the Annexin V-Fluorescein Isothiocyanate (FITC) Apoptosis Detection Kit (Nanjing Keygen, Nanjing, China). The cells were cultured at 1 × 10^6^ cells/mL and treated with rMGL (0.01, 0.1, and 1.0 U/mL). After 48 h of incubation, the cells were collected and stained with Annexin V-FITC and propidium iodide (PI) according to the manufacturer’s instructions. The untreated cells were used as the control. The rate of apoptosis was assessed using flow cytometry (Cytomics FC500 Flow Cytometer, Beckman Coulter Inc., Brea, CA, USA).

### 4.8. Statistical Analysis

The statistical analysis was performed using SPSS 13.0 software (Chicago, IL, USA). The data are presented as the means ± s.d. The differences between two groups were analyzed using the Student’s *t*-test when the data were normally distributed. If the data were not normally distributed, the differences were determined using the Wilcoxon rank sum test. The differences between multiple groups were analyzed using one-way analysis of variance (ANOVA) when the data were from normal populations and showed homogeneity of variance; alternatively, the Kruskal-Wallis test was performed. Multiple comparisons were analyzed using the Bonferroni test. All of the tests were two-sided. The differences were considered statistically significant at * *p* < 0.05, ** *p* < 0.01, and *** *p* < 0.001. 

## 5. Conclusions 

In this report, comprehensive optimization, including high-level expression, large-scale production, effective purification, desalination, and lyophilization, resulted in the cost-effective production of rMGL for therapeutic use. We investigated the anti-leukemic activities of rMGL in leukemia cell lines. The results showed that rMGL significantly inhibited the proliferation and induced apoptosis of leukemia cells in a dose-dependent manner. The data suggest that rMGL, used alone or in combination with other therapeutic methods, might be a promising drug for the treatment of patients with acute leukemia.
